# Are Changes in Consumption of “Healthy” Foods Related to Changes in Consumption of “Unhealthy” Foods During Pediatric Obesity Treatment?

**DOI:** 10.3390/ijerph9041368

**Published:** 2012-04-16

**Authors:** Shannon M. Looney, Hollie A. Raynor

**Affiliations:** Department of Nutrition, University of Tennessee, 229 Jessie Harris Building, 1215 West Cumberland Avenue, Knoxville, TN 37996, USA; Email: hraynor@utk.edu

**Keywords:** pediatric weight management, childhood obesity, fruits and vegetables, snack foods

## Abstract

Increasing fruits and vegetables (FVs), a dietary recommendation for pediatric weight management, is theorized to reduce energy intake by reducing intake of more energy-dense foods, such as snack foods (SFs). This study examined the relationship between changes in FV, SF, and energy intake in children enrolled in a 6-month, family-based behavioral pediatric weight management trial. Secondary data analyses examined dietary intake in 80 overweight (≥85th to <95th percentile for body mass index [BMI]) and obese (≥95th percentile for BMI) children (7.2 ± 1.7 years) with complete dietary records at 0 and 6 months. Participants were randomized to one of three treatment conditions: (1) increased growth monitoring with feedback; (2) decrease SFs and sugar sweetened beverages; or (3) increase FVs and low-fat dairy. With treatment condition controlled in all analyses, FV intake significantly increased, while SF and energy intake decreased, but not significantly, from 0 to 6 months. Change in FV intake was not significantly associated with change in SF consumption. Additionally, change in FV intake was not significantly related to change in energy intake. However, reduction in SF intake was significantly related to reduction in energy intake. Changing only FVs, as compared to changing other dietary behaviors, during a pediatric obesity intervention may not assist with reducing energy intake.

## 1. Introduction

Early childhood is a critical period for obesity treatment interventions because children are developing behaviors to be carried into adulthood [1]. Ideally, these learned healthy behaviors during childhood will be implemented over the lifecourse to prevent future obesity. In children, increasing fruit and vegetable (FV) consumption is one recommended dietary strategy for treating obesity [2]. To impact energy balance and help with improving weight status, increasing FVs must reduce energy intake. Solely increasing FVs in the diet with no other dietary changes will only increase energy intake [3]. Thus, for FVs to be an effective dietary strategy for pediatric weight management, increasing FVs must decrease (*i.e.*, displace) the consumption of other more energy-dense foods, thereby reducing energy intake [4]. 

Behavioral economics is a framework that provides a foundation to understand that eating behaviors can be complementary to or substitutes for each other [5,6]. Complementary eating behaviors change in the same direction (e.g., as one behavior increases, the other behavior also increases), while substitute behaviors change in opposite directions (e.g., as one behavior increases the other behavior decreases) [7]. Understanding if eating FVs substitute for eating low-nutrient-dense, high-energy-dense snack foods (SFs) (e.g., eating an apple instead of potato chips) is important in understanding the role of FVs in pediatric weight management. 

Limited research has investigated the relationship between FVs and SFs, with most research in this area conducted in adults. Of the studies conducted mixed results have been found. Several studies were unable to find a significant relationship between consumption of FVs and SFs [3,8,9] while other studies do demonstrate a relationship between intake of FVs and SFs [10,11]. For those studies suggesting that an increase in FVs is related to a decrease in energy-dense foods, analyses have examined changes in dietary intake across time. However, none of these investigations has examined the direct relationship between changes in FVs and energy-dense foods, such as SFs, to understand if they are natural substitutes. Additionally, few of the investigations have examined if an increase in FVs is also related to reductions in energy intake. 

Thus, the purpose of this secondary data analysis was to examine the relationship between changes in FVs and SFs when these food groups are individually targeted for change without an energy intake goal in children aged 4 to 9 years, who are enrolled in a 6-month, family-based behavioral pediatric weight management program. Additionally, the relationship between change in servings/day of FVs and SFs and change in energy intake from 0 and 6 months was examined. The source of this secondary data analyses is the trial conducted by Raynor and colleagues [12]. The main trial was a 6-month, family-based pediatric intervention, with 12-month follow-up, with overweight (≥85th to <95th percentile for body mass index [BMI]) and obese (≥95th percentile for BMI) children (4–9 years) randomized to one of three treatment conditions: (1) increased growth monitoring with feedback provided to families [GROWTH]; (2) decrease SFs and sugar sweetened beverages [DECREASE]; or (3) increase FVs and low-fat dairy [INCREASE] over 6 months. Results of the main trial, using intention-to-treat analyses with assessments at 0, 6, and 12 months included in the analyses, found that servings/day of FVs consumed significantly increased and servings/day of SFs consumed and energy intake significantly decreased from 0 to 6 months, with no differences occurring between the three conditions. For this investigation, it was hypothesized there would be no relationship between change in FV and change in SF intake. Additionally, due to SFs having a higher energy density than FVs, it was hypothesized reductions in SF intake would have a greater impact than increases in FV intake on reductions in energy intake.

## 2. Methods

### 2.1. Participants

Between November 2005 and September 2007 families from Rhode Island, Connecticut and Massachusetts were recruited by mailings, advertisements, community fairs, posters and flyers or through a referral from their family pediatrician/physician for a 6 month, family-based behavioral pediatric weight management randomized controlled trial. Eligibility criteria included families with an overweight (≥85th to <95th percentile for BMI) or obese (≥95th percentile for BMI) child, aged 4 to 9 years, who were not meeting at least one dietary (2 servings/day of whole fruit, 3 servings/day of vegetables, 2 servings/day of low-fat dairy products, ≤3 servings/day of low-nutrient-dense, high-energy dense SFs (*i.e.*, candies, cookies, cakes, ice cream, chips, nuts or ≤3 servings/week of sugar-sweetened beverages)) or leisure-time activity (≥60 minutes of physical activity per day or <2 hours of television per day) guideline targeted in the main trial conducted by Raynor and colleagues [12], had a parent willing to attend eight treatment sessions over six months, had a parent and child who could speak English, were not participating in another child weight management program, and who had no major psychiatric disease and no dietary or physical activity restrictions. One hundred one children met eligibility criteria and were randomized into the main trial [12]. Of the 101 children randomized, 80 met an additional eligibility criterion of having complete dietary data at both time points (0 and 6 months) and were included in this secondary data analysis. 

The study protocol was approved by the Institutional Review Board at Rhode Island Hospital (Providence, RI, USA). The main trial [12] was registered at ClinicalTrials.gov (NCT00200265). A more detailed description of trial methodology can be found in the main trial manuscript [12].

### 2.2. Procedures

Following a phone screen, interested families attended an orientation, where informed consent (and informed assent for children aged ≥ 8 years) was obtained and families were scheduled for a baseline assessment. Following completion of baseline assessments, participants were randomized to one of three treatment conditions: (1) increased growth monitoring with feedback provided to families [GROWTH]; (2) decrease SFs and sugar sweetened beverages [DECREASE]; or (3) increase FVs and low-fat dairy [INCREASE].

In the GROWTH condition, families had growth assessed at 0, 3 and 6 months and were provided with a letter stating changes in height, weight, BMI, BMI percentile and percent overweight. Families received monthly newsletters on healthy eating and leisure-time behaviors for parents which included activities for children. Both the INCREASE and DECREASE condition combined GROWTH with an 8-session (45 minutes per session) parent-only program that used parenting and behavior modification techniques to change eating behaviors that influence energy balance. Families in the INCREASE condition were encouraged to consume 2 servings/day of whole fruit, 3 servings/day of vegetables and 2 servings/day of low-fat dairy products. The DECREASE condition encouraged families to consume ≤3 servings/week of low-nutrient-dense, high-energy-dense SFs (*i.e.*, candies, cookies, cakes, ice cream, chips, nuts) and ≤3 servings/week of sugar sweetened beverages. 

### 2.3. Measures

Dependent measures were collected in a primary care or medical-school research setting by a trained researcher blinded to treatment assignment at 0 and 6 months. 

*Demographics*. Child demographic data (*i.e.*, race [American Indian or Alaskan Native, Asian, African American, Pacific Islander, White or other], ethnicity [Hispanic or Latino, not Hispanic or Latino], sex, age) were collected by a self-reported questionnaire completed by the parent.

*Anthropometrics*. Height and weight was measured using standard procedures [13]. Weight was measured to the nearest 0.1 pounds using an electronic scale (Healthometer Professional, Sunbeam Products Inc., Boca Raton, FL, USA) and height was measured, without shoes to the nearest 0.125 inches using a wall-mounted stadiometer (SECA, ITIN Scale Company, Brooklyn, NY, USA). BMI was calculated by dividing weight in kilograms by height in meter squared (kg/m^2^). BMI *z-score* was calculated by standardizing the BMI value in relation to the population mean and standard deviation for children’s age and sex [14]. Overweight (≥85th to <95th percentile for BMI) and obese (≥95th percentile for BMI) status was determined by plotting children’s BMI-for-age on the Centers for Disease Control and Prevention’s BMI percentile charts [14].

*Dietary Intake*. Dietary intake was assessed by 3-day food records (2 weekdays, 1 weekend day). Families were given an example food record with written and verbal directions about how to complete 3-day food records along with 2-dimensional food models to assist in estimating portion size. Due to the limited cognitive ability to self-report food intake parents reported intake for children under the age of 8 years [15]. The majority of food records were completed by the parent and parents assisted older children who were able to write in the food records. If a child was under the supervision of another adult, that adult was asked to complete the food record. Diaries were reviewed by a trained researcher for completion, which included confirming with parents that when the child was under the supervision of another adult, that the adult supervising the child wrote down anything that the child ate or drank and reported it to the parent. Dietary data was analyzed using the Nutrition Data System Software for Research (NDS-R) developed by the Nutrition Coordinating Center, University of Minnesota, Minneapolis, Minnesota. Only foods eaten in at least 0.125 servings were counted. Dependent variables included FV servings/day (servings/day of fruits + servings/day of vegetables), SF servings/day and energy intake. FV servings/day was the sum of all servings related to the NDS-R codes for whole low-energy-dense fruits (citrus fruit; fruit excluding citrus; avocado and similar) and whole low-energy-dense vegetables (dark-green vegetables; deep-yellow vegetables; tomato; white potatoes; other starchy vegetables; other vegetables), excluding all fruit juice, vegetable juice, fried fruits and fried vegetables. SF servings/day was the sum of all NDS-R codes related to sweet and salty snacks foods (fruit-based savory snack; vegetable-based savory snack; all crackers; all cakes, cookies, pies, pastries, Danish, doughnuts and cobblers; snack bars-refined grain; all snack chips; flavored popcorn; meat-based savory snack; nuts and seeds; yogurt-nondairy; frozen dairy and nondairy dessert; pudding and other dairy dessert; artificially sweetened pudding and other dairy dessert; chocolate and non-chocolate candy; miscellaneous dessert).

### 2.4. Statistical Analyses

A subsample (N = 80) of the families randomized (N = 101) in the main trial [12], with complete dietary records at 0 and 6-months were included in the secondary data analysis, which used a completers analyses protocol. For baseline demographic information, analysis of variance (ANOVA) and χ^2^ analyzed differences in interval/ratio and nominal measures, respectively, between treatment conditions. Change scores were calculated as 6-month measures minus baseline measures. To examine changes in FVs, SFs and energy intake that occurred over the 6-month intervention, repeated measures of analysis of covariance, with time (0 and 6 months) as the within-subjects factor and treatment condition as the covariate, were conducted. 

The relationship between change in consumption of FVs and SFs from 0 to 6 months was examined with block linear regression. Baseline child characteristics (age, sex, race, ethnicity and BMI *z-score*), were force entered into block 1, treatment condition was force entered into block 2, dietary baseline values (SF servings/day and FVs servings/day), were force entered into block 3, and change in daily servings of FVs from 0 to 6 months was force entered into block 4, with change in daily servings of SFs from 0 to 6 months as the dependent variable.

The relationship between change in consumption of FVs, SFs and energy intake from 0 to 6 months was also evaluated with a block linear regression. Baseline child characteristics (age, sex, race, ethnicity, and BMI *z-score*), were force entered into block 1, treatment condition was force entered into block 2, dietary baseline values (SF servings/day, FV servings/day, energy intake), were force entered into block 3, and change in FV and SF servings/day from 0 to 6 months were force entered into block 4, with change in energy intake from 0 to 6 months as the dependent variable. Data analysis was conducted using SPSS for Windows (version 17.0, 2009, SPSS Inc., Chicago, IL, USA) with an alpha level of α < 0.05 for significance set a priori.

## 3. Results

The main trial [12] found a significant increase in FV intake from 0 to 6 months and a significant decrease of SF and energy intake from 0 to 6 months with no significant difference between the treatment conditions. Full outcomes of the initial trial have been previously reported [12]. 

Of the 101 children in the main trial, baseline characteristics of the children included (N = 80) in this study did not significantly differ from the children excluded from this study (N = 21). Of the children included in this investigation, mean age was 7.2 ± 1.7 years, 62.5% were female, 86.3% identified as White and 17.5% as Hispanic. Children had a baseline BMI *z-score* of 2.30 ± 0.66 and a mean BMI percentile of 92.2 ± 2.0. Baseline characteristics and dietary intake of the children did not significantly differ by treatment condition (see Table 1).

**Table 1:** Baseline characteristics of children by treatment condition. (N = 80).

		GROWTH	INCREASE	DECREASE	*p*-values
		N = 26	N = 27	N = 27	
**Age, years (mean ± SD)**		6.9 ± 1.8	7.6 ± 1.6	7.3 ± 1.5	*p* = 0.33
**Sex (% female)**		61.50%	63.00%	63.00%	*p* = 0.99
**Race (%)**					*p* = 0.52
	**American Indian or Alaskan Native**	-	-	-	
	**Asian**	-	3.70%	3.70%	
	**African American**	3.80%	7.40%	14.80%	
	**Pacific Islander**	-	-	-	
	**White**	88.50%	88.90%	77.80%	
	**Other race**	-	-	-	
	**Two or more races**	7.70%	-	3.70%	
**Ethnicity (%)**					
	**Hispanic or Latino**	26.90%	11.10%	14.80%	*p* = 0.29
**Education Level of Parent (%)**					*p* = 0.20
	**High School (10–12 years)**	0.00%	18.50%	7.40%	
	**Vocational Training (beyond high school)**	3.80%	3.70%	3.70%	
	**Attended Some College**	96.20%	77.80%	89%	
**z-BMI**		2.50 ± 0.89	2.31 ± 0.52	2.11 ± 0.45	*p* = 0.10
**BMI percentile (%)**		96.4 ± 1.9	96.3 ± 1.7	95.8 ± 2.4	*p* = 0.48
**Kilocalories**		1,669 ± 464	1,644 ± 397	1,728 ± 517	*p* = 0.79
**SF servings/day**		2.1 ± 1.7	2.0 ± 1.4	2.0 ± 1.5	*P* = 0.99
**FV servings/day**		1.0 ± 0.9	0.9 ± 1.0	0.7 ± 0.7	*p* = 0.37

SD = standard deviation; z-BMI = body mass index z-score; BMI = body mass index; SF = energy dense snack food; FV = fruit and vegetable.

During the intervention period, 55.6% and 40.7% of the parents attended all sessions in INCREASE and DECREASE, respectively. With treatment condition controlled, FV consumption had a significant main effect of time with consumption increasing from 0 to 6 months (2.0 ± 1.3 servings/day *vs.* 2.8 ± 2.3 servings/day, *p* = 0.03) while SF consumption decreased over time from 0 to 6 months with a trend for significance (2.1 ± 1.5 servings/day *vs.* 1.4 ± 1.3 servings/day, *p* = 0.09). Total energy intake decreased from 0 to 6 months (1,681 ± 458 kcals *vs.* 1,511 ± 419 kcals, *p* = 0.39), but the reduction was not significant.

Change in FV intake from 0 to 6 months was not significantly related to change in SF intake from 0 to 6 months (β = 0.03, *p* = 0.73). As anticipated, baseline values of SF intake were significantly (*p* < 0.001) related to changes in SF intake in the expected direction (*i.e.*, a higher baseline value (positive value) was related to a greater reduction (negative value)). See Table 2 for the full block linear regression. Change in SF servings/day from 0 to 6 months was significantly (β = 0.63, *p* < 0.001; R^2^ = 0.617, *p* < 0.001; ∆R^2^ = 0.205, *p* < 0.001) related to change in energy intake from 0 to 6 months, while change in FV servings/day was not (β = 0.10, *p* = 0.23). As anticipated, baseline values of energy intake were significantly (*p* < 0.001) related to changes in energy intake in the expected direction (*i.e.*, a higher baseline value (positive value) was related to a greater reduction (negative value). A higher baseline intake of SF servings was significantly (*p* < 0.05) related to less of a reduction in energy intake. See Table 3 for the full block linear regression model.

**Table 2:** Block linear regression models of baseline child characteristics, treatment condition, baseline dietary variables, and change in fruits and vegetable servings per day on change in snack food servings per day.

Model (block)	Variables	β	*p*-values
	Age ^0^	0.11	*p* = 0.37
1 (I)	Sex	0.07	*p* = 0.62
	Race	0.09	*p* = 0.48
	Ethnicity	−0.05	*p* = 0.65
	BMI *z-score *^0^	0.14	*p* = 0.25
	R^2^ change = 0.035, p = 0.74; R^2^_cum_ = 0.035; F (5, 74) = 0.54, *p* = 0.74		
	Age ^0^	0.12	*p* = 0.36
2 (I & II)	Sex	0.06	*p* = 0.63
	Race	0.08	*p* = 0.54
	Ethnicity	−0.05	*p* = 0.70
	BMI *z-score *^0^	0.13	*p* = 0.31
	Treatment Condition	−0.05	*p* = 0.68
	R^2^ change = 0.002, p = 0.68; R^2^_cum_ = 0.038; F (6, 73) = 0.48, *p* = 0.82		
	Age ^0^	0.13	*p* = 0.18
3 (I, II & III)	Sex	0.06	*p* = 0.52
	Race	0.01	*p* = 0.88
	Ethnicity	0.16	*p* = 0.09
	BMI *z-score *^0^	0.02	*p* = 0.86
	Treatment Condition	−0.12	*p* = 0.17
	SF serving/day ^0^	−0.73	*p* < **0.001**
	FV servings/day ^0^	0.07	*p* = 0.47
	R^2^ change = 0.478, *p* < 0.001; R^2^_cum_ = 0.516; F (8, 71) = 9.45, *p* < 0.001		
4 (I, II, III & IV)	Age ^0^	0.12	*p* = 0.19
	Sex	0.06	*p* = 0.57
	Race	0.01	*p* = 0.89
	Ethnicity	0.16	*p* = 0.10
	BMI *z-score *^0^	0.02	*p* = 0.88
	Treatment Condition	−0.12	*p* = 0.18
	SF serving/day ^0^	−0.73	*p* < **0.001**
	FV servings/day ^0^	0.07	*p* = 0.44
	Change in FV serving/day	0.03	*p* = 0.73
	R^2^ change = 0.001, p = 0.726; R^2^_cum_ = 0.516; F (9, 70) = 8.31, *p* < 0.001		

^0^ baseline values; R^2^ change = incremental variance accounted for by each block; R^2^_cum_= variance accounted for by the entire model.

**Table 3:** Block linear regression models of baseline child characteristics, treatment condition, baseline dietary variables, change in fruit and vegetable servings per day and change in snack food servings per day on change in energy intake.

Model (block)	Variables	β	*p*-values
	Age ^0^	−0.02	*p* = 0.89
1 (I)	Sex	−0.03	*p* = 0.84
	Race	0.02	*p* = 0.87
	Ethnicity	−0.00	*p* = 0.98
	BMI *z-score *^0^	0.1	*p* = 0.45
	R^2^ change = 0.012, p = 0.97; R^2^_cum_ = 0.012; F (5, 74) = 0.19, *p* = 0.97		
	Age ^0^	−0.00	*p* = 0.95
2 (I & II)	Sex	−0.03	*p* = 0.80
	Race	−0.01	*p* = 0.91
	Ethnicity	0.02	*p* = 0.87
	BMI *z-score *^0^	0.06	*p* = 0.67
	Treatment Condition	−0.17	*p* = 0.16
	R^2^ change = 0.026, p = 0.16; R^2^_cum_ = 0.039; F (6, 73) = 0.49, *p* = 0.82		
	Age ^0^	0.15	*p* = 0.15
3 (I, II & III)	Sex	−0.07	*p* = 0.51
	Race	−0.06	*p* = 0.55
	Ethnicity	0.13	*p* = 0.21
	BMI *z-score *^0^	0.14	*p* = 0.22
	Treatment Condition	−0.16	*p* = 0.12
	SF serving/day ^0^	−0.06	*p* = 0.61
	FV servings/day ^0^	0.04	*p* = 0.72
	Energy Intake ^0^	−0.61	*p* < **0.001**
	R^2^ change = 0.373, *p* < 0.001; R^2^_cum_ = 0.412; F (9, 70) = 5.45, *p* < 0.001		
4 (I, II, III & IV)	Age ^0^	0.07	*p* = 0.44
	Sex	−0.14	*p* = 0.14
	Race	−0.07	*p* = 0.38
	Ethnicity	0.03	*p* = 0.77
	BMI *z-score *^0^	0.12	*p* = 0.17
	Treatment Condition	−0.07	*p* = 0.38
	SF serving/day ^0^	0.42	*p* < **0.05**
	FV servings/day ^0^	0.02	*p* = 0.84
	Energy Intake ^0^	−0.64	*p* < **0.001**
	Change in FV serving/day	0.1	*p* = 0.23
	Change in SF serving/day	0.63	*p* < **0.001**
	R^2^ change = 0.205, *p* < 0.001; R^2^_cum_ = 0.617; F (11, 68) = 9.97, *p* < 0.001		

^0^ baseline values; R^2^ change = incremental variance accounted for by each block; R^2^_cum_ = variance accounted for by the entire model.

## 4. Discussion

This investigation examined the relationship between changes in consumption of FVs and SFs in 80 overweight and obese children, 4- to 9-years-old, who were enrolled in a 6-month, family-based behavioral pediatric weight management randomized control trial. Results indicated there was no relationship between changes in FV and SF consumption; therefore increases in consumption of FVs were not related to decreases in consumption of SFs. Additionally, reductions in SF consumption were significantly related to reductions in energy intake. Increases in FV consumption were not related to decreases in energy intake.

The lack of a relationship between FVs and SFs is consistent with previous research in adults [3,8] and children at-risk for obesity [9]. Results of this study suggest while increasing FVs may increase the nutrient quality of the diet, as FVs are nutrient-dense foods, increasing FVs may not automatically reduce intake of high-energy-dense, non-nutrient-dense foods or energy intake. As no relationship was found between change in FVs and SFs, to produce changes in consumption of these two types of foods, they both may need to be targeted in an intervention. Thus, to optimally increase consumption of FVs and decrease consumption of SFs and reduce energy intake, interventions may need to actually target both food groups, rather than relying on only targeting one of these types of food groups during an intervention. 

While the current investigation found significant increases in FVs, it did not find significant decreases in SFs and energy, which the main trial [12] did. Differences in these findings may be due to differences in the types of analyses conducted (intention-to-treat *vs.* completers analyses), which influenced the sample size used in the analyses, and the time frame of the analyses (12 month follow-up *vs.* 6-month intervention only). However, while not significant, it is important to note that children included in this subsample did decrease SFs and energy intake. Interestingly, results also indicate that children with greater intake of SF servings at baseline had smaller reductions in energy intake. Potentially, children entering the trials with higher SF serving intake may have found it more difficult to make overall changes in the diet that could assist with reducing overall energy intake. 

Limitations of the study include use of dietary change scores in analyses, as change scores may have reduced variability. However, it is important to note that baseline values were controlled for in analyses to address regression to the mean issues related to the use of change scores. Also, energy consumed from only FVs and SFs was not able to be determined, as while the dietary software used provides food group servings consumed from combination foods, it does not provide energy intake from each individual food group that may make up a combination food. Thus, this limits the ability to examine the relationship between energy intake from food groups and overall energy intake. The study also had a relatively small sample size; but, the high rate of retention from the main trial [12] is an important strength to note. A posterior power calculation of Model 4 (Block I, II, III and IV) revealed an effect size of 0.125 (small to medium) could be detected with the sample size of this investigation (N = 80) [16]. The sample was also fairly homogenous, limiting the ability to generalize the results to overweight and obese children from underrepresented racial and ethnic groups. Analyses used completer, rather than intention-to-treat analyses, as the investigation was a secondary data analyses. Results are also limited to highly motivated, treatment seeking families. 

An additional limitation is the use of self-reported dietary measures [17]. Three recent reviews [18,19,20] of dietary assessment methodology specifically in children noted the challenges of collecting accurate dietary data and suggested that specific demographic characteristics (e.g., weight status, age, sex, ethnicity) can be related to the misreporting of dietary intake. An additional barrier to collecting accurate dietary data from children is when a child is under the supervision of another caretaker in which multiple proxy report may be necessary to capture all the items consumed in a day, such as what was done in this study [18]. 

Though several limitations exist, this is the first non-observational study to directly investigate the relationship between change in FVs and SFs across time in overweight children. As none of the conditions had recommendations to increase FVs and decrease SFs, (a combined message) this study is able to examine the natural relationship between changes in intake of these food groups when there is no prescription for energy intake during a pediatric weight management trial. This secondary data analysis provides preliminary data to help understand the relationship between changes in intake between FVs and SFs. 

## 5. Conclusions

Study outcomes suggest FVs do not displace energy-dense SFs in overweight children aged 4 to 9 years participating in a pediatric weight management intervention. To better understand how FVs and SFs impact energy balance and nutrient quality in children, future intervention investigations should explore the effects of these dietary targets alone and in combination on dietary intake and weight status to determine the most efficacious dietary strategies to treat childhood obesity. 
